# Exploration of neuron heterogeneity in human heart failure with dilated cardiomyopathy through single-cell RNA sequencing analysis

**DOI:** 10.1186/s12872-024-03739-9

**Published:** 2024-02-03

**Authors:** Yu-Hui Cui, Chun-Rong Wu, Dan Xu, Jian-Guo Tang

**Affiliations:** Department of Trauma-Emergency & Critical Care Medicine Center, Shanghai Fifth People‘s Hospital, Fudan University, No.801 Heqing Road, Minhang District, Shanghai, 200240 China

**Keywords:** Heart failure, Dilated cardiomyopathy, Neuron, Immune and inflammation response, Differentiation trajectory

## Abstract

**Objective:**

We aimed to explore the heterogeneity of neurons in heart failure with dilated cardiomyopathy (DCM).

**Methods:**

Single-cell RNA sequencing (scRNA-seq) data of patients with DCM and chronic heart failure and healthy samples from GSE183852 dataset were downloaded from NCBI Gene Expression Omnibus, in which neuron data were extracted for investigation. Cell clustering analysis, differential expression analysis, trajectory analysis, and cell communication analysis were performed, and highly expressed genes in neurons from patients were used to construct a protein-protein interaction (PPI) network and validated by GSE120895 dataset.

**Results:**

Neurons were divided into six subclusters involved in various biological processes and each subcluster owned its specific cell communication pathways. Neurons were differentiated into two branches along the pseudotime, one of which was differentiated into mature neurons, whereas another tended to be involved in the immune and inflammation response. Genes exhibited branch-specific differential expression patterns. FLNA, ITGA6, ITGA1, and MDK interacted more with other gene-product proteins in the PPI network. The differential expression of FLNA between DCM and control was validated.

**Conclusion:**

Neurons have significant heterogeneity in heart failure with DCM, and may be involved in the immune and inflammation response to heart failure.

**Supplementary Information:**

The online version contains supplementary material available at 10.1186/s12872-024-03739-9.

## Introduction

Heart failure is a kind of frequent clinical syndrome worldwide, especially in the population over the age of 65 years, which affects about 21 per 1,000 people [1]. It occurs when the heart is unable to supply enough cardiac output to maintain the metabolic demands as a result of structural or functional defects in the myocardium [2]. Heart failure carries a high morbidity and mortality, the latter is up to 50% within 5 years, resulting in many intensive care unit (ICU) admissions [3, 4]. Patients usually spend several days in the ICU, however, the cost of hospitalization represents the greatest proportion of the total cost of heart failure, and the risk of death or readmission is high [5, 6]. Heart failure hospitalization rates are rising over time, bringing severe economic pressure to patients and heavy burdens on public health care systems [7]. Unfortunately, the number of patients with heart failure is on an upward trend due to the growth of the global population and the aging population as well as the prolonged survival of heart failure patients [8–10]. Therefore, the improvement of therapeutic strategies is an urgent need for heart failure prevention and treatment. Furthermore, as widely acknowledged, there exists a strong association between dilated cardiomyopathy (DCM) and the development of chronic heart failure. Failure to receive appropriate, scientific, and efficacious treatment in DCM patients significantly increases their susceptibility to progressing toward chronic heart failure, ultimately leading to mortality. Consequently, DCM patients are frequently chosen as subjects for predictive investigations pertaining to chronic heart failure. It is evident that further investigation into DCM, acquiring additional insights, and implementing more scientifically rigorous treatment approaches for DCM will contribute to the prevention of chronic heart failure.

Single-cell RNA sequencing (scRNA-seq) technologies offer great opportunities to characterize individual cells and identify cell heterogeneity, which may contribute to enhancing the knowledge of the cellular mechanisms of heart failure [11]. Recently, Koenig et al. applied scRNA-seq to patients with chronic heart failure, and revealed the cellular landscape of the failing human heart, providing a valuable data resource for investigation in this field [12]. In the present study, we focused on the neurons in heart failure with DCM and explored their heterogeneity, differentiation trajectory, and biological functions. This study may deepen the understanding of the role of neurons in heart failure, and provide novel insight into heart failure.

## Methods

### Cell clustering analysis

The scRNA-seq profile GSE183852 was obtained from NCBI Gene Expression Omnibus, in which neuron data of patients with chronic heart failure (dilated cardiomyopathy, DCM) (HDCM1, HDCM3, HDCM4, HDCM6, HDCM8, *n* = 5) and healthy samples (HDCM5, HDCM7, *n* = 2) were extracted for further analysis. The detailed information on the samples was exhibited in Supplemental Table 1. Seurat R package [13] was utilized for cell clustering analysis. SCTransform function was used for data normalization. RunPCA function was applied for principal component analysis (PCA), and the top 20 principal components were adopted to construct the common nearest-neighbor graph. Harmony R package [14] was conducted to remove the batch effects. FindClusters and RunUMAP functions were employed respectively for cell clustering and visualization.

### Trajectory analysis

Monocle2 R package [15] was used for trajectory analysis. Genes with mean expression value > 0.1 and expression in at least 10 cells were adopted for analysis. reduceDimension function was applied for dimensionality reduction with DDRTree method. Cells were ordered by orderCells function to construct the pseudotime trajectory. Genes that varied along the pseudotime were detected by differentialGeneTest function and visualized by plot_pseudotime_heatmap function. Branch-specific genes were determined by BEAM function and visualized by plot_genes_branched_heatmap function.

### Cell communication analysis

Communication between cells and cells extremely plays a crucial role in plenty of physiological and pathological mechanisms. In this research, the identifying and illustrating alterations in intercellular signaling network (iTALK) R package [16] was employed to perform cell communication analysis among six neuron subclusters with the aim of illustrating intercellular communication signals. The analysis was carried out with a number of 4 kinds of factors, including cytokine, checkpoint, growth factor, and other factors, which can reveal the neuron subcluster interactions.

### Differential expression analysis

FindAllMarkers function from Seurat R package was used to identify the differentially expressed genes (DEGs) between neuron subclusters. The functional enrichment analysis, including Gene Ontology (GO) and Kyoto Encyclopedia of Genes and Genomes (KEGG) [17] was performed by Database for Annotation, Visualization, and Integrated Discovery (DAVID) [18].

### Construction of PPI network

Highly expressed genes involved in neural function biological processes in DCM samples were used to construct the protein-protein interaction (PPI) network through STRING [19].

### DEG validation

The dataset GSE120895, containing gene expression data from a total of 47 DCM patients with symptoms of heart failure and 8 healthy individuals, was obtained from the Gene Expression Omnibus (GEO) database as the validation set in order to compare the expression levels of DEGs we obtained in the current study between individuals with DCM and healthy controls.

### QRT-PCR validation

In order to validate the expression levels of the key genes in the PPI network in patients, samples from DCM patients and healthy volunteers were collected. Informed consent was obtained from each of all patients and volunteers, and the ethical approval for this study was also obtained from the Medical Ethics Committee of Shanghai Fifth People’s Hospital, Fudan University. After sample collection, total RNA was extracted from samples obtained from the individuals with DCM and healthy volunteers, followed by the reverse transcription procedure to obtain cDNA. Then, the qRT-PCR experiment was performed. The PCR reaction was performed with the reaction system constructed by ChamQ Universal SYBR qPCR master Mix (Vazyme Biotech) and the reaction conditions were as follows: 1 cycle of 90 °C for 30 s, 40 cycles of 95 °C for 10 s and 60 °C for 30 s. The relative expression level of each gene was calculated using the 2^−ΔΔCT^ method with GAPDH as a reference for normalization. The primer sequences can be found in Supplemental Table 2.

### Statistical analysis

The statistical analysis was employed in R (4.1.2). Wilcoxon rank-sum test was utilized for difference comparison between samples. *P*-value < 0.05 was regarded as statistically significant.

## Results

### Heterogeneity of neurons in cardiac tissue

**Fig. 1:**
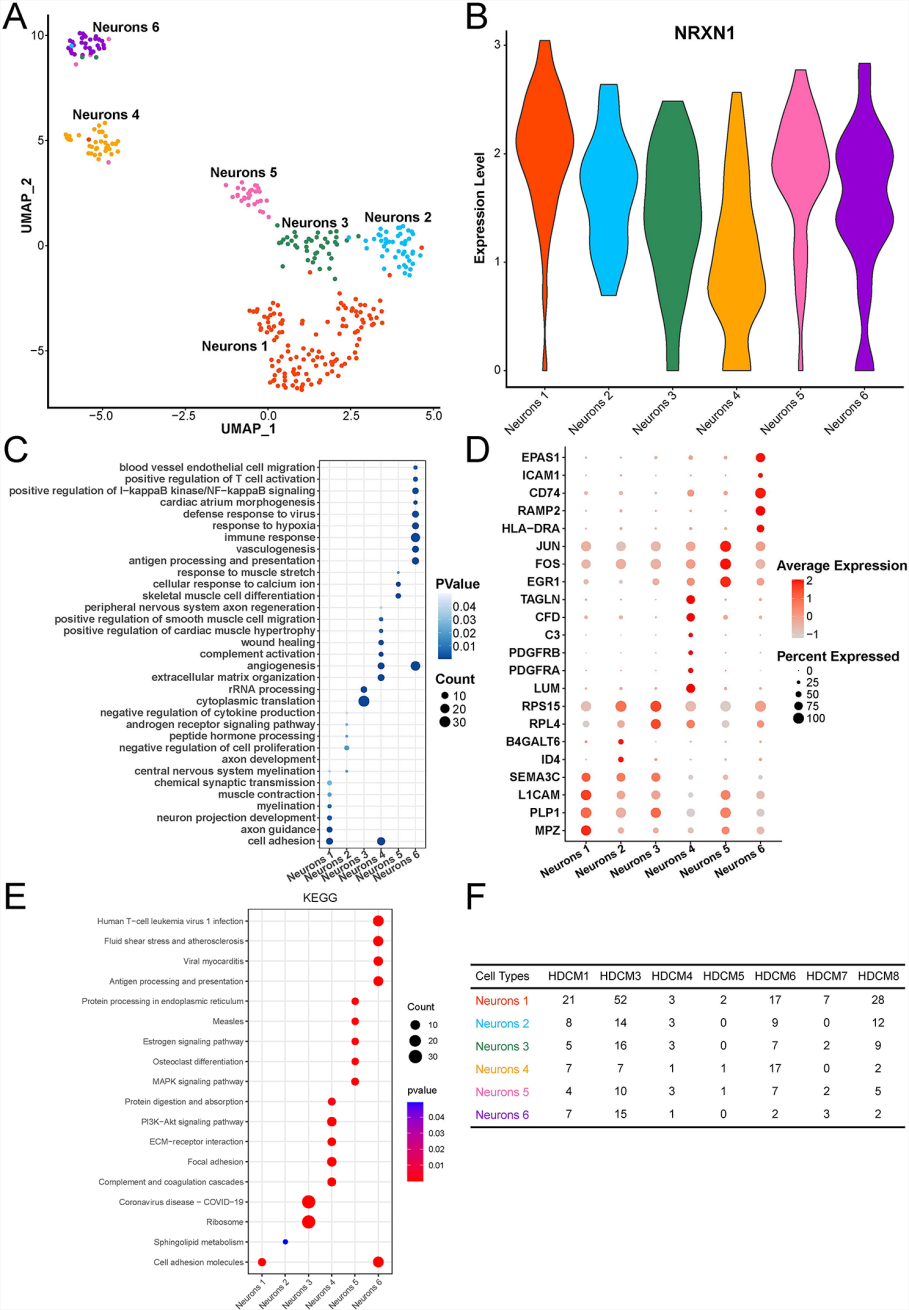
Heterogeneity of neurons in cardiac tissue. **(A)** UMAP plot of six neuron subclusters. **(B)** NRXN1 expression in six neuron subclusters. **(C)** Bubble chart of enriched biological processes by six neuron subclusters. **(D)** Bubble chart of several highly expressed DEGs in six neuron subclusters. **(E)** Bubble chart of KEGG enrichment by six neuron subclusters (KEGG data source: www.kegg.jp/kegg/kegg1.html). **(F)** Number of six neuron subclusters in each sample

In the first place, we performed the quality control analysis in order to ensure the veracity of the following research. The nCount_RNA, nFeature_RNA expression levels, and the proportions of mitochondrial genes in neurons within each sample were depicted in Figure S1. Subsequently, neurons were divided into six subclusters (Fig. 1A), all of which highly expressed NRXN1 (Fig. 1B). According to the functional enrichment analysis of cluster-enriched genes (Fig. 1D), different subclusters were associated with various biological processes (BP) (Fig. 1C). Neurons 1 mainly participated in neuron projection development, axon guidance, myelination, chemical synaptic transmission, central nervous system myelination, and cell adhesion, indicating that neurons 1 were mature neurons. Neurons 2 were primarily involved in androgen receptor signaling pathway, peptide hormone processing, and negative regulation of cell proliferation. Neurons 3 referred to cytoplasmic translation and rRNA processing. Neurons 4 chiefly took part in extracellular matrix organization, angiogenesis, complement activation, wound healing, and cell adhesion. Neurons 5 were related to skeletal muscle cell differentiation, cellular response to calcium ions, and response to muscle stretch. Neurons 6 were principally associated with antigen processing and presentation, immune response, positive regulation of T cell activation, response to hypoxia, defense response to virus, positive regulation of I-kappaB kinase/NF-kappaB signaling and angiogenesis, suggesting that neurons 6 were involved in the immune and inflammation response. The involved pathways between different subclusters were also analyzed, and the results indicated that each subcluster exhibited disparate pathway activity (Fig. 1E). Noteworthily, the KEGG enrichment results demonstrated that the pathway activity of each subcluster exhibited a high degree of consistency with the results obtained from the enrichment of BP. In each sample, neurons 1 was the most (Fig. 1F), suggesting that mature neurons accounted for the largest number of neurons in cardiac tissue.

### Differentiation trajectory of neurons and cell communication profiles

**Fig. 2:**
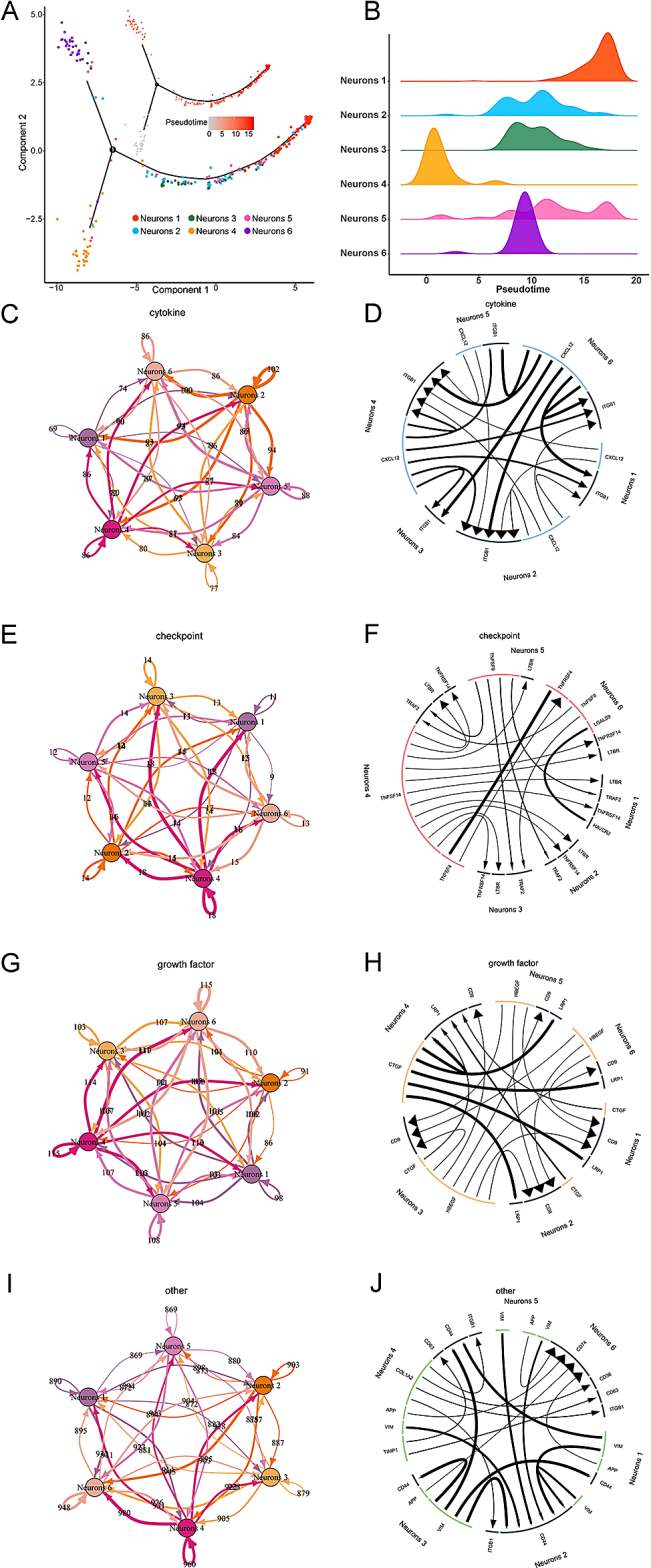
Differentiation trajectory of neurons and cell communication profiles of each neuron subcluster. **(A)** Differentiation trajectory of neuron subclusters along the pseudotime. **(B)** Ridgeline plot of six neuron subclusters along the pseudotime. **(C, D)** Cell communication profiles of cytokine module. **(E, F)** Cell communication profiles of checkpoint module. **(G, H)** Cell communication profiles of growth factor module. **(I, J)** Cell communication profiles of other module

**Fig. 3:**
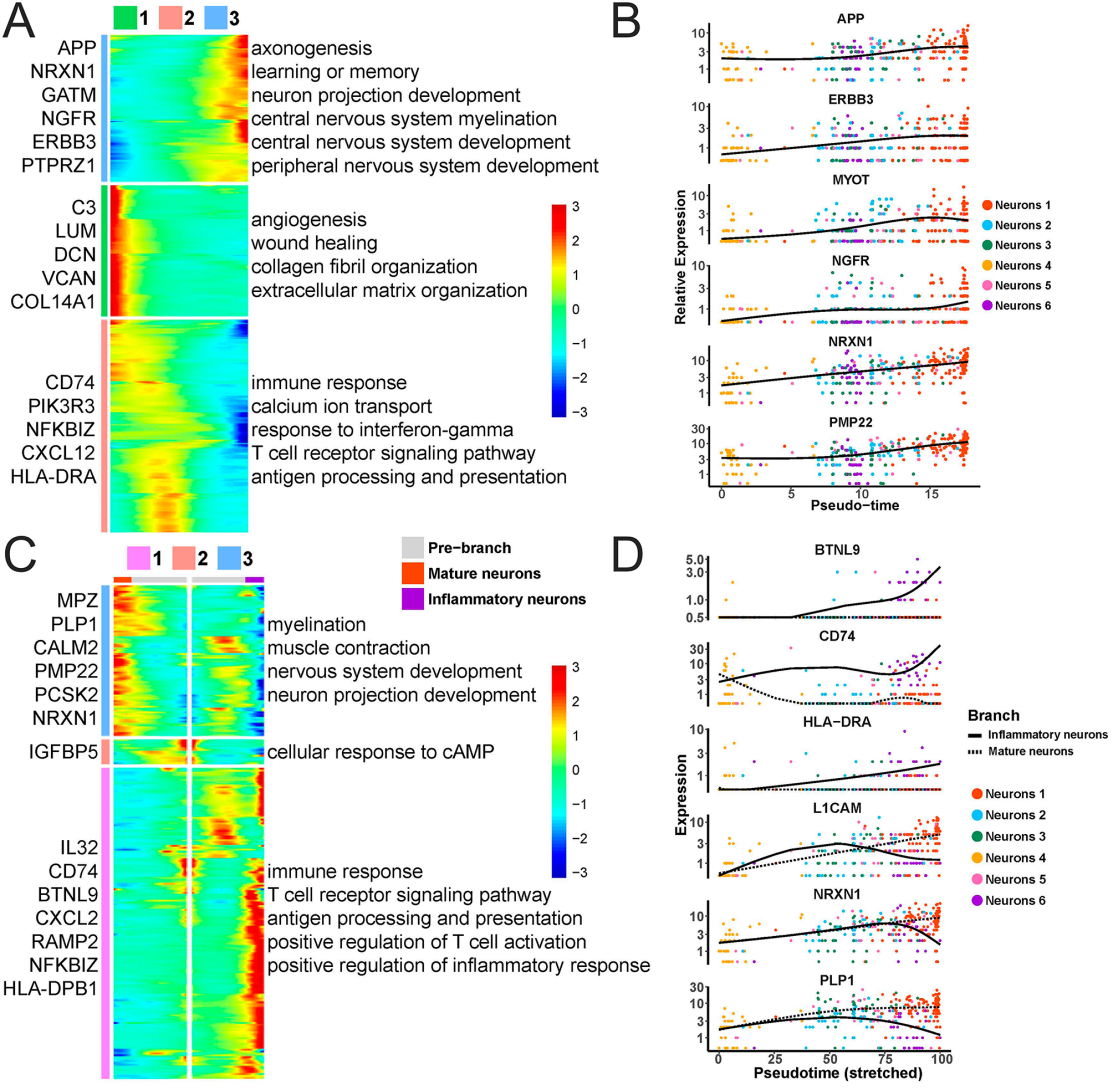
Variations in gene expression of neurons along the pseudotime. **(A)** Heatmap of enriched biological processes by genes that varied along the pseudotime. **(B)** Expression of genes that varied along the pseudotime. **(C)** Heatmap of enriched biological processes by branch-specific genes. **(D)** Expression of branch-specific genes

Starting with neurons 4, the differentiation trajectory of neurons developed two branches along the pseudotime, which respectively ended with neurons 1 and neurons 6 (Fig. 2A and B), implying that with the development of neurons, a set of neurons differentiated into mature neurons, while another set of neurons was apt to be involved in the immune and inflammation response. Neurons in two different branches were named mature neurons and inflammatory neurons, respectively. We further explored the interaction between neurons in six subclusters. In the cytokine module, ITGB1 exhibited a significant active signaling pathway across all subclusters (Fig. 2C and D). In the checkpoints module, neurons 6 displayed a higher number of active signaling pathways compared to other groups, for instance, the TNFSF9-TRAF2 receptor-ligand pair (Fig. 2E and F). Within the growth factor module, CD9 emerged as a significantly active signaling pathway in each subcluster. Additionally, HBEGF showed significant activity in neurons 3, 5, and 6 (Fig. 2G and H). Neurons 4 demonstrated a greater presence of active intracellular signaling pathways than other groups in the other module (Fig. 2I and J). Subsequently, we found that along the pseudotime, genes associated with axonogenesis, learning or memory, neuron projection development, central nervous system myelination, central nervous system development, and peripheral nervous system development were significantly up-regulated, such as APP, ERBB3, NGFR, NRXN1, and PMP22 (Fig. 3A and B). Genes associated with angiogenesis, wound healing, collagen fibril organization, and extracellular matrix organization were down-regulated, such as LUM, DCN, and VCAN. Genes associated with immune response, calcium ion transport, response to interferon-gamma, T cell receptor signaling pathway, and antigen processing and presentation exhibited an earlier increase and later decrease trend. In mature neurons, genes associated with myelination, nervous system development, and neuron projection development were significantly up-regulated along the pseudotime, such as L1CAM, PLP1 and NRXN1, whereas in inflammatory neurons, genes associated with immune response, T cell receptor signaling pathway, antigen processing and presentation, positive regulation of T cell activation and positive regulation of inflammatory response were markedly up-regulated along the pseudotime, such as BTNL9 and CD74 (Fig. 3C and D).

### Gene expression changes in neurons and PPI network construction

**Fig. 4:**
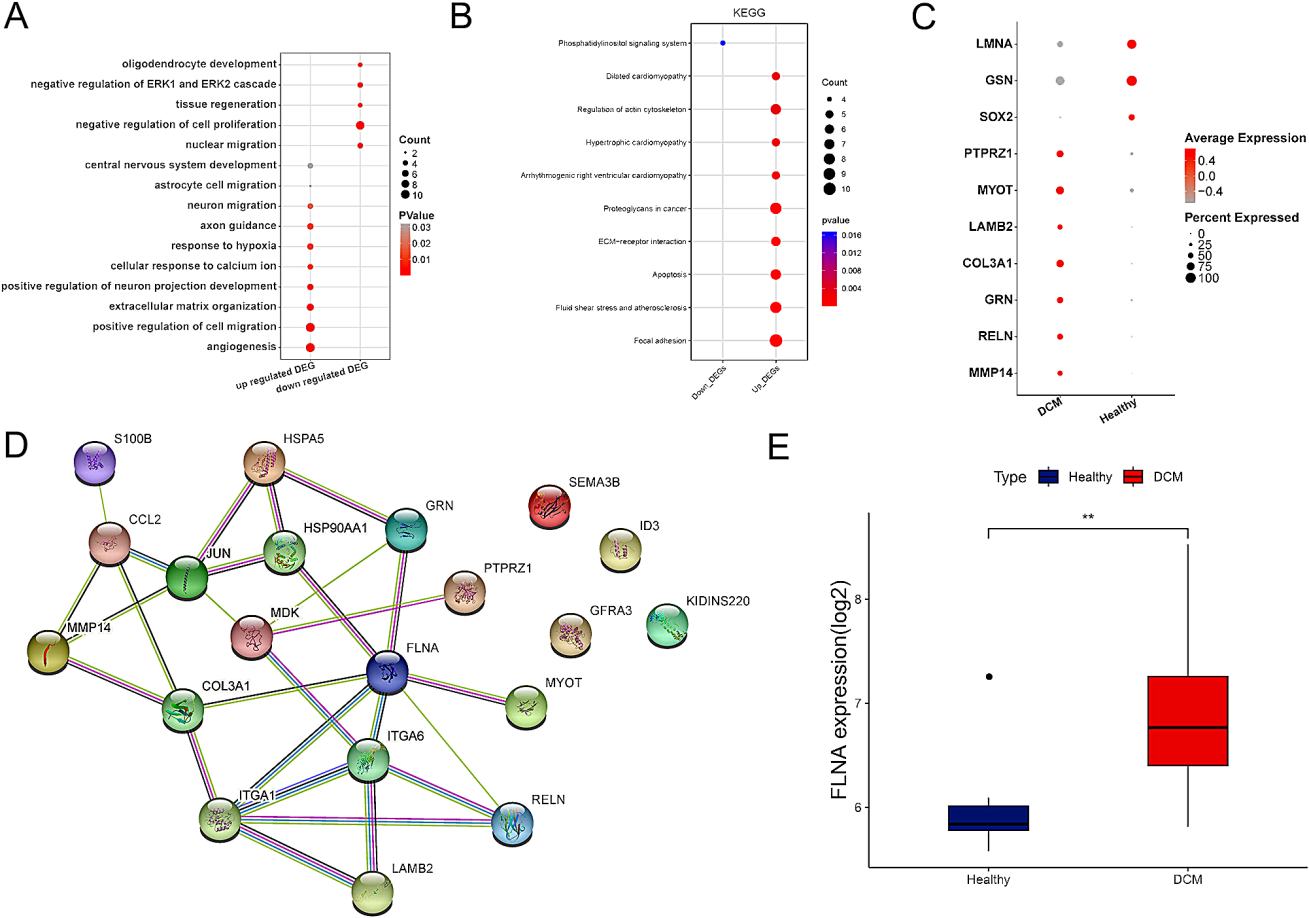
Gene expression changes in neurons. **(A)** Bubble chart of enriched biological processes by DEGs in neurons in DCM samples. **(B)** Bubble chart of KEGG enrichment by DEGs in neurons in DCM samples (KEGG data source: www.kegg.jp/kegg/kegg1.html). **(C)** Bubble chart of several highly expressed DEGs in neurons in DCM samples. **(D)** PPI network constructed by highly expressed genes in neurons. **(E)** Differential FLNA expression between DCM samples and healthy controls (** *p* < 0.01)

We explored the gene expression changes in neurons. We found that in DCM samples, highly expressed genes in neurons were mainly involved in positive regulation of cell migration, angiogenesis, extracellular matrix organization, positive regulation of neuron projection development, cellular response to calcium ions, response to hypoxia, axon guidance and neuron migration, while down-regulated genes participated in oligodendrocyte development, negative regulation of ERK1 and ERK2 cascade, tissue regeneration, negative regulation of cell proliferation and nuclear migration, and were highly involved in Focal adhesion, Apoptosis, and Fluid shear stress and atherosclerosis pathways (Fig. 4A and C). The highly expressed genes involved in neural function in neurons were used to construct the PPI network. As shown in Fig. 4D, FLNA, ITGA6, ITGA1, and MDK interacted more with other gene-product proteins, indicating that they may play important roles in neurons.

### Expression level validation of FLNA

According to the result of PPI network, we selected the gene FLNA with the highest connectivity to validate the expression level in the validation set. The result revealed a significantly elevated expression of FLNA in DCM samples compared to the healthy group (Fig. 4E).

### DEG expression levels validation by qRT-PCR

**Fig. 5:**
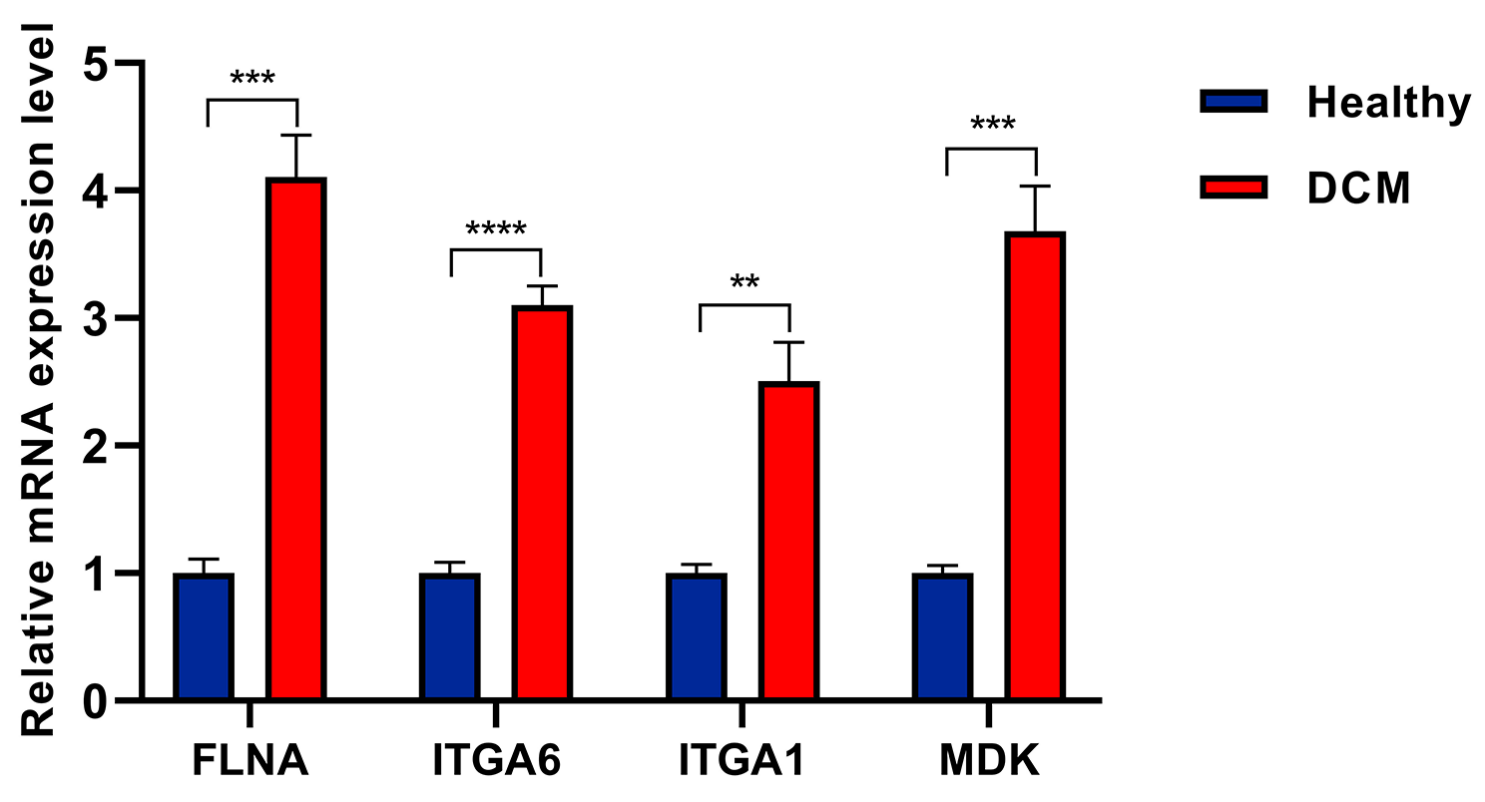
The FLNA, ITGA6, ITGA1, and MDK mRNA expression levels validation between the DCM and healthy control group by qRT-PCR. **P < 0.01; ***P<0.001; ****P < 0.0001

With the aim of validating the mRNA expression levels of DEGs obtained in the current research, qRT-PCR experiment was performed. As depicted in Fig. 5, compared to the control group, significant upregulated trends of FLNA, ITGA6, ITGA1, and MDK mRNA expression were observed in the DCM group.

## Discussion

Heart failure is a large chronic epidemic especially in the aging population, with considerable mortality and morbidity as well as frequent hospitalizations and readmissions [20]. In recent years, scRNA-seq technologies have been applied to heart failure to dissect its cell-specific information and provide the cellular landscape, helping to strengthen the understanding and promote therapeutic development of heart failure [21–23]. A recent study has conducted comprehensive analyses on bulk and scRNA sequencing data in order to clarify the cell types involved in heart failure, whose results indicated that eleven distinct cell types, including macrophage, smooth muscle cell, and dendritic cell, etc., were involved in the pathological development of DCM. Furthermore, the marker genes associated with fibroblasts, endothelial cells, dendritic cells, M1/2 macrophages, neutrophils, and smooth muscle cells were significantly enriched within the upregulated genes in DCM [24]. Similarly, there was also recent research that attempted to reveal the heart cell types through the analysis of transcriptome profiles and machine learning, which obtained similar results for cell types [25]. Nevertheless, limited relevant investigations on cardiac neurons have been conducted, and there are still significant gaps in our current knowledge of the comprehension of cardiac neurons. In this study, we focused on the heterogeneity, differentiation trajectory, and biological functions of neurons in heart failure with DCM, and revealed cell-specific gene expression patterns.

Neurons from the patients who suffered from heart failure with DCM were divided into six subclusters with high expression of NRXN1. NRXN1-encoded protein, neurexin 1, is a synaptic adhesion molecule, which is a member of the neurexin protein family. Neurexins play an important role in the vertebrate neurophysiological processes, facilitating the structure and function of synapses [26]. Previous studies have revealed that NRXN1 is associated with neurological diseases and developmental disorders, such as schizophrenia [27–29]. Zhang et al. also observed the overexpression of NRXN1 in heart failure and myocardial infarction samples and found that patients with high NRXN1 expression had an increased risk of heart failure [30]. The aforementioned statement aligns with the findings of our research that heart failure is highly associated with inflammatory neurons exhibiting a high level of NRXN1 expression. Functional enrichment analysis showed that neuron subclusters were involved in various biological processes, including synaptic formation, development and signal transmission, angiogenesis, immune and inflammation response, and so on, indicating that different kinds of neurons perform complex biological functions in heart failure. Along the differentiation trajectory, neurons developed two branches, one of which differentiated into mature neurons, while another set of neurons were involved in the immune and inflammation response. Previous studies have observed elevated levels of pro-inflammatory cytokines and chemokines in patients with heart failure [31, 32], and targeted anti-inflammatory therapy diminished heart failure-related hospitalization and mortality [33]. Therefore, according to the statement of previous research and the findings of the current study, we inferred that the inflammatory neurons with high expression of NRXN1 can trigger immune and inflammatory responses, which can contribute to the pathogenesis of heart failure [34]. Communication exists between the immune and the nervous systems [35]. Neuronal signaling can limit the immune and inflammation response by controlling cytokine release to prevent tissue damage [36]. Gene expression was changed in neurons in heart failure with DCM, and up-regulated DEGs were mainly involved in angiogenesis, extracellular matrix organization, cell migration, response to hypoxia, and so on, suggesting that neurons may enhance the activities of these biological processes as potential compensation to alleviate disease progression in the failing heart. Several genes were thought to be important in neurons through the PPI network, including FLNA, ITGA1, ITGA6, and MDK. FLNA encodes an actin-binding cytoskeletal protein and plays a role in cell-cell contact in heart and vasculature. Loss of FLNA results in vascular defects and cardiac structural and functional defects, leading to a heart failure phenotype [37, 38]. ITGA1 and ITGA6 encode proteins of integrin alpha subunit. Integrins are cell-surface receptors involved in cell-cell adhesion. The myocardial cells are anchored to the extracellular matrix by integrins, which contribute to maintaining the architecture and function of myocardium [39]. MDK encodes a heparin-binding protein, which is involved in cell growth, migration, inflammation, and angiogenesis [40]. The circulating level of MDK was increased in patients with heart failure, and MDK may be a potential marker of heart failure with DCM [41, 42].

## Conclusion

In conclusion, neurons have significant heterogeneity in heart failure with DCM and may be involved in the immune and inflammation response to heart failure. FLNA, ITGA6, ITGA1, and MDK may play an important role in neurons in heart failure with DCM.

### Electronic supplementary material

Below is the link to the electronic supplementary material.

Supplementary Material 1

Supplementary Material 2

Supplementary Material 3

## Data Availability

The datasets generated and/or analyzed during the current study are available in the NCBI Gene Expression Omnibus repository, (https://www.ncbi.nlm.nih.gov/geo/), GSE183852.
