# Supplementary material for: Exploration of neuron heterogeneity in human heart failure with dilated cardiomyopathy through single-cell RNA sequencing analysis
Source: BMC Cardiovasc Disord. 2024 Feb 3;24:86. doi: 10.1186/s12872-024-03739-9 (PMC10838417; doi:10.1186/s12872-024-03739-9)

Figure S1. The nCount_RNA, nFeature_RNA expression and the proportions of mitochondrial genes of neurons in each sample.


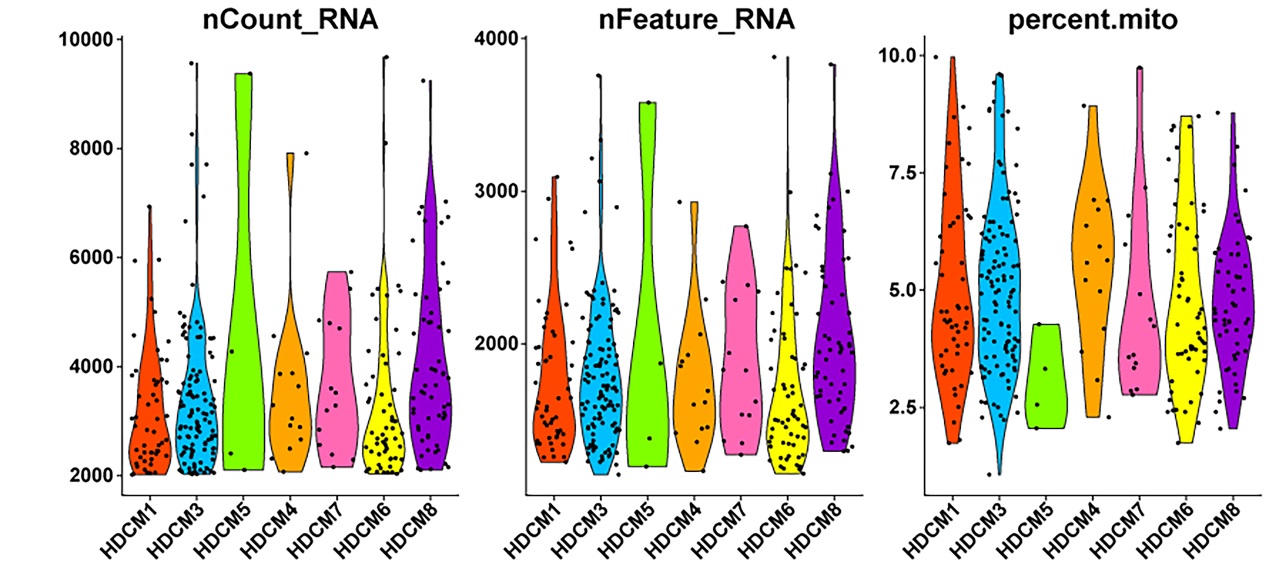

Supplement: Supplementary file 1 — Supplementary Material 1 [file 12872_2024_3739_MOESM1_ESM.docx]
