# Supplementary material for: Exploration of neuron heterogeneity in human heart failure with dilated cardiomyopathy through single-cell RNA sequencing analysis
Source: BMC Cardiovasc Disord. 2024 Feb 3;24:86. doi: 10.1186/s12872-024-03739-9 (PMC10838417; doi:10.1186/s12872-024-03739-9)
Supplement: Supplementary file 2 — Supplementary Material 2 [file 12872_2024_3739_MOESM2_ESM.docx]

**Supplemental Table 1** Detailed information on the samples for analyses. (0 represents no, while 1 represents yes)

| Sample | Age | Sex | Condition | Race | Ethnicity | BMI | Etiology of HF | Pre-Op EF | Pre-Op  CO (L/min) | Arrhythmias | Type | Valve disease | Type VD | HTN | Diabetes | CKD | Smoking |
| --- | --- | --- | --- | --- | --- | --- | --- | --- | --- | --- | --- | --- | --- | --- | --- | --- | --- |
| HDCM1 | 55 | M | DCM | AA | NH | 19.7 | NICM | 20 | 2.96 | 1 | VT | 1 | Mild MR, TC, PR | 1 | 0 | 1 | 1 |
| HDCM3 | 23 | F | DCM | AA | NH | 23.53 | NICM | 19 | 3.21 | 0 | N/A | 1 | Mod MR, Mod TR | 0 | 0 | 0 | 1 |
| HDCM4 | 74 | M | DCM | W | NH | 24.74 | NICM | 14 | 3.67 | 1 | afib, a flutter, NSVT | 1 | AS | 0 | 0 | 1 | 1 |
| HDCM5 | 63 | F | Healthy | W | NH | 25.51 | NICM | 29 | 3.61 | 1 | VT, afib | 1 | Sev MR | 0 | 0 | 0 | 1 |
| HDCM6 | 60 | M | DCM | W | NH | 21.3 | NICM | 23 | 3.17 | 0 | N/A | 1 | Mild MR, severe TR, mild PR | 0 | 1 | 0 | 1 |
| HDCM7 | 63 | M | Healthy | AA | NH | 28.19 | Healthy | 50 | 6.6 | 0 | N/A | 0 | N/A | 1 | 0 | 0 | 1 |
| HDCM8 | 38 | M | DCM | W | NH | 27.49 | Healthy | 63 | 6.4 | 0 | N/A | 0 | N/A | 1 | 0 | 0 | 1 |
