# Supplementary material for: Exploration of neuron heterogeneity in human heart failure with dilated cardiomyopathy through single-cell RNA sequencing analysis
Source: BMC Cardiovasc Disord. 2024 Feb 3;24:86. doi: 10.1186/s12872-024-03739-9 (PMC10838417; doi:10.1186/s12872-024-03739-9)
Supplement: Supplementary file 3 — Supplementary Material 3 [file 12872_2024_3739_MOESM3_ESM.docx]

Supplemental Table 2 Primer sequences

| Genes | Primer direction | Sequences |
| --- | --- | --- |
| FLNA | F  R | CAGCTCGAGTGCTTGGACAAT  GTCAGCGAAGAGGATGTTGATGT |
| ITGA6 | F | AGGTCTGGCCTCTTCATTTGG |
| ITGA1 | R  F  R | TCTTGCCACCCATCCTTGTT  TCCATTGCCCCTGTACAAGAA  CTGTTGGAACCATCCAGCACTA |
| MDK  GAPDH | F  R  F | GTGCCCTGCAACTGGAAGA  GCACCCCAGTTCTCAAACTTGT  GAAGGTGAAGGTCGGAGTC |
|  | R | GAAGATGGTGATGGGATTTC |
